# Effectiveness and cost-effectiveness of sector-independent treatment coordination for people with substance-related disorders following an online assessment (ASSIST): study protocol for a randomized controlled trial

**DOI:** 10.1186/s13063-022-06343-4

**Published:** 2022-06-01

**Authors:** Annabel S. Mueller-Stierlin, Jeanette Röhrig, Christian Goetzl, Michael Krausz, Jutta Lehle, Elke Prestin, Vanessa-Emily Schoch, Lorenz Sutter, Jean Westenberg, Maurice Cabanis

**Affiliations:** 1 Department of Psychiatry II, Ulm University, Bezirkskrankenhaus Günzburg, Günzburg, Germany; 2 Institute of Epidemiology and Medical Biometry, Ulm University, Ulm, Germany; 3 Centre for Mental Health, Clinic for Addiction Medicine and Addictive Behavior, Klinikum Stuttgart, Stuttgart, Germany; 4 Department of Psychiatry, University of British Columbia, Vancouver, Canada

**Keywords:** Substance use disorder,, Substance-related problems, Addiction,, Sector-independent treatment coordination,, Online assessment,, Treatment allocation,, e-Health, Satisfaction,, Cost-effectiveness

## Abstract

**Background:**

The implementation of person-centred, need-oriented and flexible care for people with substance-related problems is often insufficient, in large part due to the complexity of addiction support services among different providers. A standardized online assessment and subsequent sector-independent treatment coordination could provide individuals with more appropriate services, thereby making better use of individual services and leading to a more effective addiction support system as a whole. The aim of this study is to determine the effectiveness and cost-effectiveness of sector-independent treatment coordination following an online assessment, in comparison with the current standard of care and treatment process in Germany.

**Methods:**

The sample size of this randomized, controlled trial has been set to a total of 400 participants with substance-related problems. Participants living in Stuttgart, Germany, will be randomly allocated to (1) the intervention group with immediate online assessment and subsequent sector-independent treatment coordination (ASSIST) or (2) the waitlist group. Participants in the waitlist group will initially remain in usual care and only be provided with the online assessment 6 months later. Short-term effects (over 2 months) and medium-term effects (over 6 months) of ASSIST will be compared between the intervention and the waitlist groups. The primary outcome is improved treatment satisfaction. Secondary outcomes include improved subjective quality of life and empowerment, reductions in patients’ substance use, unmet needs and illness-related clinical and social impairment. Health economic evaluation as well as quantitative and qualitative process evaluations will be conducted.

**Discussion:**

The results of this study are expected to provide information on whether sector-independent treatment coordination following an online assessment contributes to improved health care service provision for people with substance-related problems. This randomized controlled trial will help identify facilitators and barriers to the sustainable implementation of a cross-sectoral care concept in substance abuse services.

**Trial registration:**

German Clinical Trial Register DRKS00026996. Registered on 29 October 2021

## Administrative information

Note: The numbers in curly brackets in this protocol refer to the SPIRIT Checklist item numbers. The order of the items has been modified to group similar items (see http://www.equator-network.org/reporting-guidelines/spirit-2013-statement-defining-standard-protocol-items-for-clinical-trials/).

**Table:** 

Title {1}	Effectiveness and cost-effectiveness of sector-independent treatment coordination for people with substance-related disorders following an online assessment (ASSIST): study protocol for a randomized controlled trial
Trial registration {2a and 2b}.	German Clinical Trial Register DRKS00026996 Item 2b is met.
Protocol version {3}	29/10/2021 Version 1.1
Funding {4}	This study is funded by the Innovation Fund of the Federal Joint Committee (G-BA), grant number 01NVF19016 (ASSIST).
Author details {5a}	Dr. Annabel S. Mueller-Stierlin^1,2^, Dr. Jeanette Röhrig^3^, Christian Goetzl^1^, Prof. Dr. Michael Krausz^4^, Jutta Lehle^1^, Dr. Elke Prestin^1^, Dr. Vanessa-Emily Schoch^3^, Lorenz Sutter^3^, Jean Westenberg^3,4^, Dr. Maurice Cabanis^3^ ^1^ Department of Psychiatry II, Ulm University, Bezirkskrankenhaus Günzburg, Günzburg, Germany ^2^ Institute of Epidemiology and Medical Biometry, Ulm University, Ulm, Germany ^3^ Centre for Mental Health, Clinic for Addiction Medicine and Addictive Behavior, Klinikum Stuttgart, Stuttgart, Germany ^4^ Department of Psychiatry, University of British Columbia, Canada
Name and contact information for the trial sponsor {5b}	Dr. Maurice Cabanis, Klinikum der Landeshauptstadt Stuttgart, Clinic for Addiction Medicine and Addictive Behavior, Prießnitzweg 24, 70374 Stuttgart, mail: m.cabanis@klinikum-stuttgart.de, phone: 0049 - 711 278-22245
Role of sponsor {5c}	The sponsor initiated the trial and was involved in designing the study. Representatives of the sponsor—MC, JR, VES, LS—belong to the trial steering committee. The sponsor will not be involved in the collection, management, or analysis of the data. But the sponsor will be involved in the interpretation of the findings and the preparation of the report for publication, though the decision to submit the report for publication is up to the principal investigator (AMS).

## Introduction

### Background and rationale {6a}

In Germany, 1.6 million people are diagnosed with an alcohol use disorder [1] and an estimate of 1.9 million people with prescription drug abuse and misuse [2]. Gomes de Matos et al. [3] have shown that 19% of participants have problematic consumption related to alcohol, followed by 5.2% related to prescription drugs and 1.6% related to illegal substances [4].

The global burden of disease from opioid use disorders is estimated to be about 12 million disability-adjusted life years, accounting for 70% of the burden of all drug use disorders and representing a 22.3% increase between 2005 and 2015 [5–7]. In international comparisons, Germany is ranking third (after the USA and Canada) for the levels of consumption of narcotic drugs with 28,842 daily doses/million [6]. Depending on the study and sample, the 1-year incidence of opioid prescription abuse for 2012–2014 ranges between 0.008 and 5.0% [7, 8]. Concerning the addiction support system, there have been many known issues and challenges over the years, including poor identification of substance use disorders, inadequate networking between service providers, abstinence as a compulsory treatment goal and unclear strategies to assign people who use substances (PWUS) to services.

According to Mann [9], less than 5% of people in Germany who need treatment for substance use disorder actually receive addiction-specific therapy. Instead, primary medical care which is not specific to addiction is frequently used [10]. The majority of individuals with alcohol use disorder have reported that they have been in contact with primary medical care services at least once in the past year. Furthermore, patients are often only reached in an advanced stage of the disorder, after a long history of the unaddressed or untreated disease. According to Mann, less than half of the people with alcohol use disorder are diagnosed correctly [9].

In addition, the fragmentation of the German health care system results in insufficient networking between service providers and a lack of integrated interventions for PWUS [11, 12].

The system too often focuses on abstinence, neglecting other goals such as reduction of substance use, risk reduction and social support [13]. In other European countries, like for example Great Britain or the Netherlands, controlled drinking with the goal of restricted consumption is already part of the standard of care [14].

In addition, it is often unclear which criteria practitioners use to decide the most appropriate intervention for an individual. It is not only the presence of a diagnosis that should determine treatment mode, but rather the individual patient needs and specific characteristics [15].

Moreover, the concept of empowerment requires that decision-making becomes a joint process which includes both the (professional) expertise of the practitioner and the (personal) expertise of the patient [16]. This means that the appropriateness of an intervention from the professional point of view is no longer the only relevant criterion. Instead of this, plans of action have to be negotiated and finally agreed upon by both the practitioner and the patient. The very challenging situation of the current SARS-CoV-2 pandemic continues to be the cause of an increase in alcohol consumption in some subgroups. Among others, this includes people who have lost their jobs due to the pandemic or have suffered financial losses [17]. It seems essential to provide a range of care and counselling services due to the nature of this global crisis. These are to be based on digital standards and enable optimal care for PWUS, despite the restriction of personal contact. Beyond the pandemic, online assessments are a promising strategy for engaging previously hard-to-reach groups and a larger number of individuals in care [18].

To overcome the above-mentioned shortcomings in the current system of care for PWUS, the implementation of a Regional Competence Centre could play a decisive role. Starting with an online assessment tool (based on the Measurements in the Addictions for Triage and Evaluation (MATE) [19]), sector-independent, indication-based treatment recommendations (also called Treatment Assistant) can be developed, according to treatment intensity or level of care (LOC) as well as the individual needs and treatment goals (shared decision-making). Several downstream processes can then be conducted on the online platform, including treatment allocation, documentation and revisions of the plan of treatment as needed. Such a system of care, a sector-independent treatment coordination for people with substance-related disorders following an online assessment (ASSIST), should first be evaluated in comparison with the current system in terms of implementation, effectiveness and cost-effectiveness.

### Objectives {7}

The overarching aim of this project is to transform the support system for persons with high-risk substance use and substance use disorder by implementing easily accessible, sector-independent, indication-based treatment which is planned and decided upon cooperatively by practitioners and patients. Specifically, the aim of this study is to determine the effectiveness and cost-effectiveness of sector-independent treatment coordination following an online assessment in comparison with care as usual in a randomized controlled trial (RCT) design with the waitlist group. Implementation outcomes of the complex intervention will be assessed by means of quantitative and qualitative data. Finally, validity and intern consistency as reliability will be analysed for the German version of the MATE-Q used during the intervention and the EPAS used as the outcome in PWUS.

### Trial design {8}

This is a pragmatic randomized controlled superiority trial. In the Stuttgart region, 400 patients with substance-related problems will be randomly assigned to prompt treatment coordination based on an online assessment or to the waitlist control group, with a 1:1 allocation. Participants in the waitlist group will initially remain in usual care and only be provided with the online assessment 6 months later. Treatment progress will be recorded at four measurement points in time, which are 2 months apart and executed over 6 months. In the waitlist group, a fifth measurement point occurs 2 months after the online assessment. The primary outcome is improved treatment satisfaction over 6 months. Furthermore, short-term and medium-term effects will be evaluated in terms of subjective quality of life and empowerment, patients’ substance use, unmet needs and illness-related clinical and social impairment. A health economic evaluation from the perspective of the German national economy will also be conducted.

Process evaluation will be based on study data gathered by research associates, and routine data will be collected by service providers. The process evaluation is complemented by qualitative data on the collective view of PWUS, counsellors, service providers, and other stakeholders in the context of focus groups or expert interviews. The results include information on collective assessment, as well as perceived barriers and facilitators with the intervention.

## Methods: participants, interventions and outcomes

### Study setting {9}

The project is to be carried out in Stuttgart, Germany, in cooperation with service providers of the local addiction support system. The list of local service providers is available online (https://www.stuttgart.de/medien/ibs/Web_Rat_und_Hilfe_171130.pdf). The Stuttgart addiction support system is characterized by a myriad of specific counselling and treatment structures among different providers. Diagnostic standards and treatment principles vary, sometimes significantly, due to the diversity of principles and goals in the treatment process, from low-threshold assistance to hospital treatment and rehabilitation. The complexity of the health care system, as well as different providers and their responsibilities within it, has led to insufficiently implemented person-centred, need-oriented and flexible help for PWUS. Despite the existence of effective services, there is much room for improvement, and the coordination and communication between such services and treatment programmes are to the detriment of PWUS [16].

### Eligibility criteria {10}

The inclusion criteria for participants are as follows:

- 18 to 65 years old
- Resident of Stuttgart
- Reporting substance-related disorder or hazardous consumption
- Insured with a German statutory health insurance

The exclusion criteria for participants are as follows:

- Homelessness
- Insufficient German language skills
- Presence of severe neurological or psychiatric disease resulting in severe impairment of cognition
- Acute mental crisis
- Acute danger to self or others
- Serious criminal involvement in the past 5 years
- Forensic psychiatric treatment
- Suspension of entitlement to benefits pursuant to § 16 SGB V

### Who will take informed consent? {26a}

Research associates will provide individuals with verbal and written information regarding the study, who will then be asked to provide written informed consent agreeing to participate in this study.

### Additional consent provisions for collection and use of participant data and biological specimens {26b}

Informed consent includes consent to use participant data captured on the online platform by participants themselves and implementers (Regional Competence Centre and local service providers).

## Interventions

### Explanation for the choice of comparators {6b}

Participants in the waitlist group will receive care as usual only during the first 6 months of the study (until t3; waitlist period). The intervention, starting with the online assessment and an appointment at the Regional Competence Centre, will not take place before t3 is completed. The current standard of care in Stuttgart is mainly provided by hospitals, day clinics, rehabilitation centres, primary physicians, psychiatrists, psychotherapists, psychologists, social workers and addiction counselling centres. A variety of non-medical vocational and psychosocial services are provided by vocational rehabilitation centres, community mental health care centres, and various residential and nursing facilities. In Germany, 86% of the treatment and counselling services are delivered in the outpatient setting. Of these, 83% are drug and addiction counselling services [20]. In general, these services are sought out by PWUS, either on their own accord or through support from others (relatives, friends, colleagues or current professional supporters). Likewise, there is no universally applied assignment of patients to individual service providers based on treatment goals. There is also no standardized, structured or inter-agency communication, let alone a common database for all addiction-specific service providers.

### Intervention description {11a}

The intervention is intended to optimize health care planning and service coordination for people with substance-related disorders.

By means of an online assessment, participants will initiate the diagnostic process in a self-determined manner. Subsequently, employees at the Regional Competence Centre will (1) provide treatment recommendations derived from the online assessment according to the appropriate LOC as well as individual goals of the participant [21]; (2) discuss recommendations with the PWUS, revise the treatment plan accordingly, until a mutual agreement has been reached; (3) facilitate and tailor the allocation of services as well as the coordination of all those involved in the care of the participant; and (4) accompany the participant in their treatment trajectory, continuously adjusting it in cooperation with the participant if and when necessary. An online platform will assist the staff at the Regional Competence Centre with tasks such as networking, documentation, treatment allocation and coordination in the Stuttgart addiction support system.

The online assessment is based on the MATE-Q, the self-report form of the MATE that was originally developed in the Netherlands in the context of national restructuring processes of the addiction support system [22]. The MATE is based on the bio-psycho-social health model of the International Classification of Functioning (ICF). In its comprehensive form, the MATE is a semi-structured interview procedure consisting of validated measurement instruments. The MATE can be used for the allocation to specific levels of care (LOC, according to intensity following the stepped care approach) with the help of an allocation guideline or a decision tree [22]. Feasibility, validity and reliability studies have been conducted in the Netherlands [22]. The MATE was also translated and validated for the German-speaking countries [23], adapted to the allocation guideline of the German system [24] and tested using a randomized controlled trial [25, 26].

Within 1 week of completing the online assessment, a 1- to 2-h appointment will take place at the Regional Competence Centre in order to (1) complete the MATE by adding more data to the MATE-Q data collected online, (2) apply the allocation guideline to determine the appropriate LOC, (3) ask service users for their individual goals and needs, (4) solve discrepancies regarding the recommended LOC and the personal goals and needs of participants, (5) prepare an individual Treatment Assistant based on these findings and (6) prepare a report for the service users and their current or future service providers.

The Treatment Assistant is made available to service users in both paper-based and digital forms via the online platform. The staff at the Regional Competence Centre jointly with the online platform provides support, if necessary, in making referrals to the appropriate services included in the individual Treatment Assistant. Another important task of the online platform is to provide real-time feedback on free treatment capacities from the cooperating service providers. In this way, the Treatment Assistant can be adapted so that further treatment can be carried out promptly if necessary, without having to put up with long waiting times. Appointments with the service providers are arranged by the staff at the Regional Competence Centre or by service users themselves via the online platform.

After completion of each treatment step, at the latest after 2 months, the staff of the Regional Competence Centre will contact the service user to find out about their current treatment status (e.g. whether the Treatment Assistant was implemented as agreed) and to make any necessary adjustments to the Treatment Assistant, especially in terms of changes to treatment goals, in cooperation and by means of shared decision-making with the user.

All the staff at the cooperating service providers of the addiction support system in Stuttgart will be trained in the use of the Treatment Assistant (e.g. regarding information about which treatment steps have been planned, which steps have already been implemented, which steps are planned for the future). This is intended to facilitate interfaces and reduce barriers and communication difficulties within the support system (e.g. seamless transition of patients between different service providers). In addition, the cooperating service providers are asked to report the end of the treatment period to the Regional Competence centre via the online platform. If a patient deviates from the Treatment Assistant over time, the participant and the service provider should give feedback to the Regional Competence Centre via the online platform so that the Treatment Assistant can be reviewed and adjusted jointly by the staff of the Regional Competence Centre and the participant. On the online platform, a service user record is created including data from the online assessment and the Treatment Assistant, supplemented with data from various sources (service user, service providers and Regional Competence Centre). The sovereignty of the data lies with the service user, who can choose which type of information gets passed on to which service provider at each treatment step.

### Criteria for discontinuing or modifying allocated interventions {11b}

If the participant asks for discontinuing or modifying the allocated interventions or the professional recommends a modification after consultation with the participant or if the technical preconditions are not given or the participant does not respond anymore, the intervention will be discontinued or modified.

### Strategies to improve adherence to interventions {11c}

Prior to the implementation of the Regional Competence Centre and of the online platform, a survey with the staff of cooperating service providers at the addiction support system in Stuttgart (*n* = 92) was conducted, as well as qualitative studies with PWUS (*n* = 13), service providers (*n* = 13) and other stakeholders (*n* = 6) to assess local requirements, wishes and concerns, with the aim of improving the appropriateness and user-friendliness of the intervention. Therefore, core statements and results of the sub-studies with practical relevance for the implementation were exchanged with the intervention team to allow for the adaption of the intervention.

All the staff at the Regional Competence Centre and cooperating service providers in Stuttgart is trained in order to ensure the high fidelity of the intervention, especially in the development and the use of the Treatment Assistant.

The individual and flexible approach used to develop (and modify, if necessary) the Treatment Assistant, in partnership with the participant, is expected to improve adherence to the intervention. The online platform is characterized by an attractive, intuitive design that offers a high degree of user-friendliness and enables a positive user experience improving the user-adherence.

### Relevant concomitant care permitted or prohibited during the trial {11d}

There are no restrictions regarding concomitant care during the trial.

### Provisions for post-trial care {30}

As the intervention relies mainly on the coordination of standard care services in Stuttgart, the participants can use most of the services (with an exception for the online platform and the services of the Regional Competence Centre) even beyond the end of the study. A long-term implementation of the Regional Competence Centre and of the Online Platform beyond the study period is attempted, too. As it is not expected that anybody will suffer harm from trial participation, specific post-trial care is not required. Nevertheless, if harm is caused to the participant, the service providers in charge of the participant’s care will outline and coordinate the appropriate measures, in partnership with the participant.

### Outcomes {12}

#### Primary outcome

The primary outcome is the mid-term change in satisfaction with substance-related care over 6 months. With the increasing emphasis on patient engagement and continuous quality improvement in mental health care, evaluation has shifted to measuring the performance of a treatment programme in terms of treatment satisfaction [27]. Treatment satisfaction represents an important indicator of patient benefit [28]. The German short version of the Client Satisfaction Questionnaire (CSQ-8) [29], called the ZUF-8 Questionnaire [30], will be used every 2 months (t0, t1, t2, t3 and t4 if applicable).

#### Secondary outcomes

The secondary outcomes are participant’s empowerment, quality of life, perceived needs, functioning, clinical and psychological impairment and severity of substance use. These will be assessed at t0, t1, t3 and t4. Further secondary outcomes are frequency of substance use, craving, somatic symptoms, depression and anxiety. These are measured twice, at t0 and t3. The tools used to measure the secondary outcomes are as follows:

- Assessment of Empowerment in Patients with Affective and Schizophrenic Disorders (EPAS) [31]: The EPAS questionnaire measures empowerment as the patient’s perceived possibilities to control his or her own life on five dimensions: daily living, social relationships and sexuality, treatment participation, self-efficacy and self-esteem. This self-report questionnaire has 33 items and five additional items each for patients who are employed and for patients with minor children.
- Euro Quality of Life - 5 Dimensions (EQ-5D) [32]: The EuroQol (EQ-5D-5L) is used to assess health states as a basis for determining quality-adjusted life years (QALY).
- World Health Organization Quality of Life - Short Version (WHO-QoL-BREF) [33]: The WHOQOL-BREF captures the substance user’s subjective quality of life on the dimensions of physical health, mental well-being, social relationships and environmental conditions. The WHOQOL-BREF is a self-report questionnaire with 25 items.
- Camberwell Assessment of Need - European Version (CAN) [34]: The CAN is a rating instrument to record PWUS’s perceived needs and the extent to which they are met, covering 23 areas.
- World Health Organization Disability Assessment Schedule (WHODAS 2.0) [35]: The 12-item WHODAS 2.0 is a valid, reliable self-report instrument for the assessment of functioning and generic disability [36].
- Health of the Nation Outcome Scale (HoNOS) [37, 38]: The HoNOS is a 12-item rating instrument to assess clinical and psychosocial impairment of people with mental disorders in four categories: behaviour, impairment, symptoms and social functioning.
- Severity of Dependence Scale (SDS) [39]: The SDSs are 5-item screening questionnaires measuring the severity of dependence on alcohol and drugs.
- Timeline Follow Back [40, 41]: The calendar method, called Timeline Follow Back, is used to get an estimate of the participant’s daily patterns and frequency of substance use over 7 days.
- Mannheim Craving Scale (MaCS) [42]: The MaCS Questionnaire, consisting of 12 items and 4 additional items, measures obsessive-compulsive symptoms in the context of substance abuse and dependence.
- Somatic Symptom Scale (SSS-8) [43, 44]: The 8-item Somatic Symptom Scale (SSS-8) was used as a brief, patient-reported outcome measure of somatic symptom burden.
- Patient Health Questionnaire (PHQ-4) [45]: The Patient Health Questionnaire is a 4-item measure to screen for depression.
- Generalized Anxiety Disorder Scale (GAD-7) [46]: The Generalized Anxiety Disorders Scale is a 7-item screening tool for detecting generalized anxiety disorder.
- Perceived Stress Scale (PSS-10) [47, 48]: The 10-item Perceived Stress Scale was used to assess perceived psychological stress.

#### Health economic outcomes

The Client Sociodemographic and Service Receipt Inventory (CSSRI-EU) [49] will be used to record service use. Costs from the perspective of the national economy are estimated by multiplying the units of services used by the identified costs of those units and the addition of indirect costs due to work incapacity. Based on the data in the CSSRI-EU, treatment pathways including delay until allocation as well as adherence to, discontinuation of and switches between services can be presented.

#### Implementation factors and outcomes

Comprehensive process evaluation will be based on routine data, surveys and qualitative studies to identify implementation processes as well as barriers and facilitators, especially with regard to the service user involvement, use of digital technologies in healthcare or interagency and interprofessional collaboration.

Therefore, a uniform routine documentation is implemented at the Regional Competence Centre and on the online platform. In addition, fidelity criteria will be assessed, and feedback data will be collected with the staff and service users via the online platform: During the intervention, routine data will be collected from service users, employees of the cooperation partners and the Regional Competence Centre. The routine data include, e.g., basic psychiatric recording data, treatment duration data in the Treatment Assistant and changes in wishes and goals of the service users during the treatment process. Also, the overall satisfaction with the Regional Competence Centre will be collected from the clients and the employees of the cooperation partners.

Subjective and collective views of service users, service providers and other stakeholders will be gathered through qualitative interviews and focus groups at the end of the implementation phase. A flexible guideline will be used, which covers the following main topics: (1) acceptance, content evaluation and practicality of the online and face-to-face services; (2) subjective assessment of the effects in terms of treatment retention and satisfaction; and (3) barriers and facilitators for active use of the coordination platform by patients, counsellors and treatment providers. The findings will help to further tailor the treatment coordination to the needs of users and to identify facilitators and barriers to sustainable implementation or scale-up.

### Participant timeline {13}

Participant recruitment will start on November 1, 2021, and is to be completed within 12 months. The individual participation period in the study will be 6 (intervention group) or 8 months (waitlist group). There will be four or five data collections at 2-month intervals (t0, t0 + 2 months [t1], t0 + 4 months [t2], t0 + 6 months [t3] and if applicable t0 + 8 months [t4]). The follow-up windows are defined as ± 4 weeks for the intended time point. The last data collection of the last study participant should be completed by July 1, 2023.

The following study schemes depict the time schedule of enrolment, interventions and assessments (Table 1).

**Table 1:**
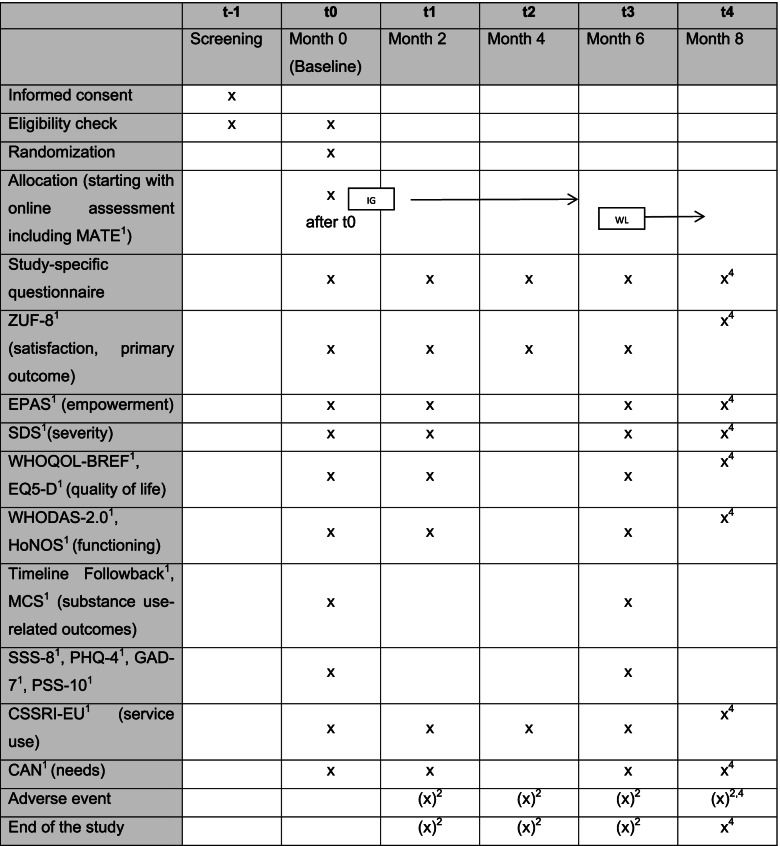
Time schedule

Process evaluation (routine data, additional polls and qualitative studies) are not depicted in the time schedule

^1^Standardized instrument

^2^If needed

^3^In the intervention group only

^4^In the waitlist group only

### Sample size {14}

Assuming a simplified statistical model (repeated measures design with within (time) × between (group) interaction, 2 groups, 4 time points) and a dropout rate of 40%, a sample size of 400 subjects allows the detection of an effect with a moderate effect size (Cohen’s *f* = 0.25 [50]) for the primary outcome (change in satisfaction (ZUF-8 Score) over 6 months) at a significance level of *p* < 0.05 with a power ≥ 0.90, as calculated by means of G*Power 3.1 [51].

### Recruitment {15}

The study will be introduced to PWUS via various channels (e.g. service providers, posters, flyers, press releases and online posts). Information is provided in personal conversations and/or via flyers and posters.

If a person is interested, an initial screening of the inclusion and exclusion criteria will take place, and if the substance user is eligible for participation, an appointment with a research associate will be scheduled. The initial screening can be made by telephone, online or in person.

## Assignment of interventions: allocation

### Sequence generation {16a}

Randomized assignment to the two study groups—intervention and waitlist—with a 1:1 allocation will be conducted using the ROM software [52] in order to avoid selection bias. A computer-generated randomization schedule with permuted blocks of random sizes will be used.

### Concealment mechanism {16b}

The independence of the group allocation from the data collection and analysis processes is guaranteed as randomization will be independently carried out by the Institute of Epidemiology and Medical Biometry at Ulm University.

All participants fulfilling the eligibility criteria and signing the informed consent form will be randomized. For this, research associates will schedule the baseline assessment (t0) and request randomization by e-mail from the Institute for Medical Biometry and Epidemiology, Ulm University, Germany. The randomization form includes the participant ID, approval of informed consent and eligibility. The requesting research associate will obtain a response by mail within one working day. Allocation disclosure will occur after the baseline assessment (t0) is completed.

### Implementation {16c}

The Institute for Medical Biometry and Epidemiology at Ulm University will generate the allocation sequence. The research associates will enrol participants, initiate randomization, collect baseline data, and assign the participants to the intervention group or the waitlist group. Thereafter, the research associate will provide access information for the online assessment to the participants of the intervention group and the study participants will complete the online assessment on their own. If required, the study participants will complete the online assessment at the premises of the Regional Competence Centre. Directly after the online assessment, a prompt appointment (best within 7 days) with the staff at the Regional Competence Centre is scheduled via the online platform. Participants in the waitlist group will receive access information for the online assessment only after completion of the third follow-up assessment (t3). This will be ensured by having two different code sets which grant access to the online platform. Only those codes provided to the intervention group are valid directly.

## Assignment of interventions: blinding

### Who will be blinded {17a}

Due to the nature of the intervention, blinding of the participants and service providers is not possible. Furthermore, it is not possible to blind the research associates, as they would be unblinded at the latest when collecting the data for the process evaluation. The same applies to all researchers involved in data monitoring and data management. Thus, only the researcher performing the main data analysis will be blinded until the main analysis has been completed.

### Procedure for unblinding if needed {17b}

Unblinding is not needed as participants, service providers and research associates will be unblinded to treatment allocation.

## Data collection and management

### Plans for assessment and collection of outcomes {18a}

Data collection will be carried out by the staff of Ulm University, who will be trained accordingly before the start of the study and will follow uniform standard operating procedures. Data collection will be carried out in face-to-face meetings at the Regional Competence Centre, the facilities of the Stuttgart addiction support system or somewhere else, or in online meetings, depending on the participants’ preferences and current contact restrictions due to the SARS-CoV-2 pandemic. At the beginning of each appointment, the evaluation staff will conduct a short admission interview to determine whether data collection is currently possible or whether there is a state of acute intoxication or acute crisis present.

As a standard, data is collected directly online via SoSci Survey (SoSci Survey GmbH, Munich). If electronic data collection is not possible, the data collection will be paper-based and transferred electronically within 1 week.

Study dropouts and serious adverse events will be documented on standardized forms.

The coordinator of the evaluation and research associates will check the quality of data collection during semiannual monitoring visits, focusing on the eligibility of included study participants as well as on the completeness and plausibility of the collected data. Furthermore, serious adverse events must be documented appropriately.

Routine data will be provided by participants via the online platform and collected by the Regional Competence Centre staff.

### Plans to promote participant retention and complete follow-up {18b}

In general, the research staff will try to fulfil the participants’ preferences regarding format (online or face-to-face), date and time when making the appointment for data collection.

The research staff will be in close contact with the study participants. At the end of the interviews, an appointment will be made for the next follow-up, which will take place after 2 months. Two weeks before this date, the participants will be reminded about the appointment. If necessary, an alternative appointment will be arranged. If the participants do not show up for the agreed appointment, they will be contacted again with the offer of an alternative appointment.

If it is not possible for the participant to be interviewed at the given time (e.g. due to a crisis or inpatient stay), the respective follow-up assessment may be omitted. Then, the research staff will attempt to arrange another appointment at the next possible time.

Participants will receive the following incentives: €10 at t0, €15 at t1, €20 at t2 and €25 at t3, and participants in the waitlist group receive €30 on t4. If applicable, they will be reimbursed for their expenses for travelling to the location of the interview.

If a participant declares his or her wish to drop out of the study, the time and the reasons for dropping out will be documented.

All deviations from the intervention protocol (including non-use and discontinuation) will be traceable using the data of the online platform and will be documented by the staff at the Regional Competence Centre.

### Data management {19}

Data will primarily be collected using the survey software SoSci Survey (SoSci Survey GmbH, Munich). When filling in the questionnaire electronically, the answer categories are pre-defined, and warnings appear if individual items have not been answered.

All collected study data will be regularly requested and stored on servers at the Department for Psychiatry and Psychotherapy II of Ulm University. Pseudonymized routine data will be provided as a .xlsx file by the intervention team at the end of the intervention phase and will be merged with study data by intervention identifier (ID). Data processing using software packages like SPSS, version 25and SAS version 9.4 will include electronic data checks for plausibility and completeness as well as the derivation of additional variables (such as scores) for analysis.

### Confidentiality {27}

The collection, storage and analysis of the study data will be carried out in compliance with the relevant data protection regulations. All personal data will be pseudonymized during the collection, by replacing the study participants’ personal identifying data with identifiers (study ID for study data and intervention ID for routine data). The index tables for the assignment of personal data and identifiers will be access-protected and stored separately from the data.

The research associates responsible for the data collection will be the only persons with access to the major index table with personal data, study identifiers and intervention identifiers.

Prior to enrolment in the study, the potential study participants will be informed orally and in writing about the study’s aims, nature, scope and implications as well as about the data protection regulations within the study by the research staff of Ulm University. The potential study participants will be given sufficient time to consider their participation and to ask questions. By signing the informed consent form, the potential study participant will agree to both, participating in the study and agreeing to the data protection regulations.

The study results will be published anonymously, making sure that identification of individual participants is not possible. The study data will only be transferred anonymously to third parties, including the sponsoring team and service providers of the addiction support system in Stuttgart.

The study data will be archived on servers at the Department for Psychiatry and Psychotherapy II of Ulm University.

### Plans for collection, laboratory evaluation and storage of biological specimens for genetic or molecular analysis in this trial/future use {33}

No biological specimens for genetic or molecular analysis will be collected in this trial.

## Statistical methods

### Statistical methods for primary and secondary outcomes {20a}

The analysis of the primary and secondary outcomes will follow the intention-to-treat (ITT) principle. Mixed models with fixed time effects and time × group interaction effects (fixed: time (4 or 2 time points), group (2 groups), time × group interaction; random: patient) will be applied. The time by group interaction will reflect the intervention effect.

The cost-effectiveness ratios from the perspectives of the national economy will be determined using the net benefit method.

Furthermore, for the waitlist group, the changes in the primary and secondary outcomes criteria during the waiting period (from t0 to t1) and after the assessment and treatment coordination (from t3 to t4) are compared using a paired *t*-test.

Implementation of sector-independent treatment coordination starting from an online assessment is evaluated mainly using descriptive methods (relative frequencies and measures of agreement). These methods are also used to show the extent to which treatment recommendations based on the online assessment and the interview at the Regional Competence Centre differ from each other and the extent to which actual treatment pathways match the recommendations. Fit is classified as less than adequate (treatment intensity lower than recommended), adequate (treatment intensity as recommended) and more than adequate (treatment intensity higher than recommended). The factors that influence fit and the extent to which fit affects the outcomes are also examined.

The statistical significance level will be set at *p* < 0.05. The statistical programmes IBM SPSS 25, STATA 15 and SAS 9.4 will be used for statistical analysis.

### Interim analyses {21b}

No interim analyses are planned.

### Methods for additional analyses (e.g. subgroup analyses) {20b}

Substance-specific effects (legal vs. illegal substances) will be examined using mixed-effect models extended by the interaction effect of time × treatment × substance group.

An in-depth analysis should reveal effects in subgroups (e.g. legal or illegal substance use, individuals with a little or a lot of experience with services or PWUS with specific treatment goals).

### Methods in analysis to handle protocol non-adherence and any statistical methods to handle missing data {20c}

In addition to the main analyses and in order to assess the maximal intervention efficacy in ideal conditions, a per-protocol (PP) approach will be followed to analyse the primary outcome. The criteria for the PP population will be defined in the statistical analysis plan prior to data analysis and may include the following:

- Incomplete online assessment and/or Treatment Assistant.
- The time between study entry and appointment at the Regional Competence Centre is too long.
- Infrequent use of the online platform by participants in the intervention group or no use after the appointment at the Regional Competence Centre.
- Follow-up assessment (t3) being far outside the initially planned time periods, i.e. more than 2 months before or after the time point that is 6 months after baseline assessment (t0).
- Long-term absence (more than 4 weeks) from Stuttgart and the local addiction support system during the intervention.

No imputation of missing data will be needed for the main analysis via mixed linear models. For the sensitivity analysis, missing values will be handled using the fully conditional specification (FCS) method [53]. The number of imputations will be chosen based on the portion of missing values. Then, each of the completed datasets will be analysed using the proposed statistical method. According to Rubin’s rule [54], the final result will be the average result of all completed datasets.

### Plans to give access to the full protocol, participant-level data and statistical code {31c}

The full protocol (including the data-management and statistical-analysis plans) and the statistical code will be available on request from AMS. All data for which there is no conflict with data-protection regulations will be published with open access.

## Oversight and monitoring

### Composition of the coordinating centre and trial steering committee {5d}

MC and JR will oversee the whole project including the intervention and the communication between the funding agency and the project team. LS will coordinate the project management and the implementation of the intervention.

The evaluation team of Ulm University comprises ten persons: AMS, as principal investigator and biostatistician, will oversee the entire study process including data management and data analysis. EP will coordinate the evaluation and will provide peer perspective in the conduct of the study and the interpretation of findings. They will form the evaluation team together with two senior researchers (TB and RK) and seven junior researchers (CG, JL, TBA).

The trial-steering committee consists of AMS and TB from the evaluation centre, as well as MC and JR as representatives of the sponsoring team.

### Composition of the data monitoring committee, its role and reporting structure {21a}

The evaluation team of Ulm University, that is independent from the sponsor, will monitor the data. Given the non-adaptive design, the rather short study period and the low probability for serious adverse events (SAEs), no interim analyses are needed and the need for preterm determination of the study is unlikely. No independent data monitoring committee was established for these reasons.

### Adverse event reporting and harms {22}

No adverse events caused by the intervention are expected. Nevertheless, all SAEs occurring during the trial observed by the research associates, the service providers or reported by the participant, no matter the study arm they belong to, will be recorded in the case report form in SoSci Survey. The study participants will be requested (if possible) to immediately inform the responsible research associate about SAEs. The occurrence of SAEs will be reported, at the latest, at the next follow-up assessment.

### Frequency and plans for auditing trial conduct {23}

There are no plans for independent trial auditing. But, periodic audit procedures for study conduct and intervention will take place by the coordinating centre, the evaluation team and the trial steering committee. For this purpose, there is a sophisticated communication plan that includes 1st separate weekly team meetings of the coordinating team including the intervention team and the evaluation team, 2nd bi-weekly meetings of the project managers of the coordinating team (LS) and the evaluation team (EP) and 3rd quarterly meetings of the trial-steering committee. These meetings provide the opportunity to evaluate the progress of the study and obtain information about potential problems. There will be an annual report to the funding body on the course of the study, the obtained findings and the impact of deviations from the study protocol and of incidents during trial conduct.

Several procedures are implemented by the coordinating team to control the quality of the interventions within the local addiction support system as follows:

- Regular meetings with the project team
- Discuss and practise different the procedures and situations with the project team
- Regular check-ups of the technical components
- Ensuring of the technical support
- Regular contacts with local cooperation partners
- Informing and supporting the teams of the care providers
- Contacting study participants by the Regional Competence Center if information or feedback is missing
- Assessment of the fidelity criteria
- Regular information of the cooperation partners about the current status of the study
- Regular information of the city council

Regarding data integrity, a research assistant with training in data supervision will verify the consistency and completeness of data each month. In addition, this research assistant will conduct semi-annual monitoring visits with research assistants responsible for data collection. The research assistant will assure that data are accurate and in agreement with any paper source documentation used, verify that informed consent for study participation has been properly obtained and documented, confirm that participants entered into the study meet inclusion and exclusion criteria, verify that study procedures are being conducted according to the standardized operating procedures and monitor serious adverse event reporting.

### Plans for communicating important protocol amendments to relevant parties (e.g. trial participants, ethical committees) {25}

Any modifications to the protocol which may impact the study’s conduct or the potential benefit to the participants or which may affect participant safety will require a formal amendment to the protocol. Important protocol amendments must be submitted to local ethical committees and be communicated to all directly involved parties, specifically the sponsoring team, the employees at the Regional Competence Centre, the Institute for Epidemiology and Medical Biometry at Ulm University, the funder and if needed the participants and service providers. The entry at the German Clinical Trial Register DRKS00026996 will be updated accordingly.

### Dissemination plans {31a}

The dissemination plan follows best-practice recommendations and aims to engage with PWUS, service providers and the public to disseminate the findings across private and public fields. All collaborators are encouraged to contribute to the dissemination of findings. The research results will be disseminated in peer-reviewed journals as well as during international and national conferences. In addition, findings will be disseminated widely to all stakeholders via public platforms and social media. A project report in plain language will be sent to all participants and will be accessible on the project’s website.

## Discussion

ASSIST is a complex intervention that aims to overcome well-known issues and challenges of the German addiction support system (e.g. poor identification of substance users, inadequate networking between service providers, abstinence as a compulsory treatment goal, and unclear strategies to assign PWUS to addiction-specific treatment options) by combining established innovative approaches in mental health care. Based on an online assessment and sector-independent, indication-based and goal-specific treatment recommendations, PWUS will be assigned to appropriate services of the Stuttgart addiction support system. The individual and patient-centred Treatment Assistant, developed with input from the PWUS in the Regional Competence Centre, will be displayed on an online platform that facilitates treatment monitoring and collaboration across the different service providers.

This protocol sets out the methods for evaluating the impact of ASSIST as an extension to the existing care as usual in Stuttgart. Stuttgart is the capital of the federal state of Baden-Württemberg and is characterized by a large, diverse and professional addiction support system [55]. This RCT with a waitlist design and embedded process evaluation will help to gain knowledge about the effectiveness and cost-effectiveness of ASSIST, but also about implementation outcomes and determinants.

A major strength is the full-coverage cooperation with service providers of the entire region of Stuttgart, enabling treatment coordination across a wide range of services and levels of care. However, the SARS-CoV-2 pandemic and related contact restrictions have complicated the initiation and strengthening of cooperation during the pre-implementation phase. As ASSIST is a complex intervention relying on good networking with local service providers, the implementation success depends on each individual team member at local service providers and their willingness to engage themselves in the project. Thus, the low contact in the pre-implementation phase might impede the recruitment of study participants and the use of the Treatment Assistant by employees of cooperating facilities.

Another strength is that the effectiveness will be evaluated independently of the service providers by Ulm University conducting a RCT, the gold standard in effectiveness evaluation. Moreover, the prospective waitlist design allows for the evaluation of intra-individual changes under both conditions. On top of that, a comprehensive set of quantitative and qualitative data will be gathered to gain deep insights into implementation processes. As some of the scales to be used have not been validated yet in comparable conditions (e.g. the German version of the MATE-Q used during the intervention and the EPAS used as an outcome in PWUS), validity and intern consistency as reliability will be analysed as part of this study.

If the results of the current study are positive, it is planned to scale up ASSIST to other communities in Germany. The development of ASSIST more broadly will benefit from the extensive knowledge gained during the trial in terms of facilitators and barriers to sustainable implementation of a cross-sectoral care concept in substance use services. Finally, this randomized controlled trial will generate the first major body of evidence on the effectiveness and cost-effectiveness of sector-independent treatment coordination for people with substance-related disorders following an online assessment in Germany.

## Trial status

Protocol version 4.0, September 10, 2021 (second amendment)

Recruitment started on November 1, 2021, and is expected to last for 12 months until November 1, 2022.

## Abbreviations

ASSIST: Sector-independent treatment coordination for people with substance-related disorders following an online assessment
CAN: Camberwell Assessment of Need - European Version
CSQ-8: Client Satisfaction Questionnaire
CSSRI-EU: Client Sociodemographic and Service Receipt Inventory – European Version
DHS: Deutsche Hauptstelle für Suchtfragen
EPAS: Assessment of Empowerment in Patients with Affective and Schizophrenic Disorders
EQ-5D: Euro Quality of Life - 5 Dimensions
FCS: Fully conditional specification
GAD-7: Generalized Anxiety Disorder Scale
G-BA: Federal Joint Committee
HoNOS: Health of the Nation Outcome Scale
ICF: International Classification of Functioning
ID: Identifier
ITT: Intention-to-treat
LOC: Level of care
MaCS: Mannheim Craving Scale
MATE: Measurements in the Addictions for Triage and Evaluation
PHQ-4: Patient Health Questionnaire
PP: Per-protocol
PSS-10: Perceived Stress Scale
PWUS: People who use substances
QALY: Quality-adjusted life years
RCT: Randomized controlled trial
SAEs: Serious adverse events
SDS: Severity of Dependence Scale
SSS-8: Somatic Symptom Scale
WHODAS 2.0: World Health Organization Disability Assessment Schedule, Version 2.0
WHO-QoL-BREF: World Health Organization Quality of Life - Short Version
ZUF-8: Questionnaire of Treatment Satisfaction
